# The G‐protein‐coupled chemoattractant receptor Fpr2 exacerbates neuroglial dysfunction and angiogenesis in diabetic retinopathy

**DOI:** 10.1096/fba.2020-00034

**Published:** 2020-09-15

**Authors:** Ying Yu, Shengding Xue, Keqiang Chen, Yingying Le, Rongrong Zhu, Shiyi Wang, Shuang Liu, Xinliang Cheng, Huaijin Guan, Ji Ming Wang, Hui Chen

**Affiliations:** ^1^ Eye Institute Affiliated Hospital of Nantong University Nantong China; ^2^ Cancer and Inflammation Program Center for Cancer Research National Cancer Institute at Frederick Frederick MD USA; ^3^ CAS Key Laboratory of Nutrition, Metabolism and Food Safety Shanghai Institute of Nutrition and Health Chinese Academy of Sciences Shanghai China

**Keywords:** diabetic retinopathy, Fpr2, glial cell, neovascularization

## Abstract

Diabetic retinopathy (DR) as a retinal neovascularization‐related disease is one of the leading causes of irreversible blindness in patients. The goal of this study is to determine the role of a G‐protein‐coupled chemoattractant receptor (GPCR) FPR2 (mouse Fpr2) in the progression of DR, in order to identify novel therapeutic targets. We report that Fpr2 was markedly upregulated in mouse diabetic retinas, especially in retinal vascular endothelial cells, in associated with increased number of activated microglia and Müller glial cells. In contrast, in the retina of diabetic *Fpr2* ^−/−^ mice, the activation of vascular endothelial cells and glia was attenuated with reduced production of proinflammatory cytokines. Fpr2 deficiency also prevented the formation of acellular capillary during diabetic progression. Furthermore, in oxygen‐induced retinopathy (OIR) mice, the absence of Fpr2 was associated with diminished neovasculature formation and pathological vaso‐obliteration region in the retina. These results highlight the importance of Fpr2 in exacerbating the progression of neuroglial degeneration and angiogenesis in DR and its potential as a therapeutic target.

**Abbreviations**
DR: diabetic retinopathy
FPR: formyl peptide receptor
GPCR: G‐protein‐coupled chemoattractant receptor
NF‐κB: nuclear factor κB
OIR: oxygen‐induced retinopathy
RMEC: retinal microvascular endothelial cells
VEGF: vascular endothelial growth factor

## 1 INTRODUCTION

Ischemia‐ and chronic inflammation‐induced neovascularization is the main clinical feature of diabetic retinopathy (DR) which is the leading cause of blindness in patients.^1^, ^2^ However, the use of inhibitors for vascular endothelial growth factor (VEGF) for the treatment of DR achieved disappointing results.^3^ Therefore, it is imperative to understand the relationship between angiogenesis and inflammation in the pathogenesis of DR to develop novel and more effective therapies against retinal neovascularization.

Evidence suggests that retinal glial cells contribute to the pathogenesis of DR,^4^ which displays a unique spatial arrangement of glial cells intercalating between vasculature and neurons to facilitate the uptake of glucose by glia and subsequent transfer of energy to neurons.^5^ Vaso degeneration is accompanied by neuroglial abnormalities resulting in the loss of ganglion cells.^6^ In rat diabetic retina, Müller cells exhibit electron dense nuclear granulations and nuclear chromatin dispersion, in association with the accumulation of glycogen, dense bodies, and lysosomes cytoplasmic.^7^ Müller cells secret growth factors such as VEGF contributing to vascular leakage, inflammation, and neovascularization in the development of DR.^8^ In contrast, astrocytes display functions different from Müller cells under hyperglycemia, in which astrocytes show a decreased density and as a major cell population in the optic nerve, are responsible for remodeling the structure of lamina cribrosa.^9^ Compared to Müller cells, microglial cells are more dynamic in nature and respond rapidly to changes in microenvironment.^10^ Reactive microglia are active participants in DR and may promote the progression of the disease to a proliferative state.^11^ In the early stage of DR, perivascular microglia are hypertrophic, and surround dilated new vessels in a feature of microglial perivasculitis.^12^

Cellular responses to microenvironmental changes are dependent on membrane ‘sensors’ including a variety of cell surface receptors mediating cell chemotaxis and proliferation. Formyl peptide receptors (FPRs) belong to the G‐protein‐coupled chemoattractant receptor (GPCR) family and are expressed by a variety of immune cells, such as neutrophils, macrophages, and microglia to mediate cell recruitment and mediator release in response to pathogen‐ and tissue‐derived chemotactic agonists.^13^ FPRs also regulate the expression and secretion of angiogenic factors by malignant tumor cells and tumor stem cells.^14^, ^15^, ^16^ FPR2 (mouse Fpr2) is one of the FPR family member, expressed by a various cell types, such as phagocytes, fibroblasts, and endothelial cells. Resting murine microglial cells express low levels of Fpr2, which was upregulated by pathogen‐ and host‐derived molecules associated with inflammatory and innate immune responses. Fpr2 on activated microglial cells become highly responsive to chemotactic agonists produced in the central nervous system.^17^, ^18^ Fpr2 also plays an important role in the angiogenic process in diabetic states, as shown by the observation in which an FPR2 agonist peptide stimulates the formation of von Willebrand factor‐positive capillary, suggesting that it stimulates angiogenesis thus may have therapeutic potential in wound healing.^19^ The capacity of Fpr2 to mediate angiogenesis is also shown by stimulation of the formation of endothelial tubules and aortic ring by an agonist.^20^ It is also reported that Fpr2 mediates inflammation and angiogenesis in PDR vitreous in response to peptide agonists, suggesting the receptor as a target for PDR therapy.^21^

Our previous study revealed that Fpr2 on Müller glial cells was upregulated by high glucose and participated in the pathological process of DR.^22^ This promoted us to further examine the role of Fpr2 in two models of DR, one recapitulating the early vascular and neuroglial degenerative stage and the other representing the ischemia‐induced neovascular stage. Here we report that Fpr2 accelerates neuroglial and vascular degeneration culminating in neovascularization.

## 2 MATERIALS AND METHODS

### 2.1 Reagents

Anti‐FPR2 [GM1D6] (ab26316), anti‐IBA1 [EPR16589] (ab178847), and anti‐CD31 [MEC 7.46] (ab7388) monoclonal antibodies; anti‐GFAP (ab7260), anti‐Vimentin (ab45939), anti‐ki67 (ab15580), and anti‐Collagen IV (ab19808) polyclonal antibodies were from Abcam (Cambridge, UK). Anti‐CRAMP (R‐170) polyclonal antibody was from Santa Cruz (Santa Cruz, CA). Isolectin GS‐IB4 (i21412) was from Invitrogen (Carisbad, CA). Mouse CRAMP was synthesized by New England Peptide LLC (Gardner, MA). Alexa Fluor 488 and Alexa Fluor 568 were from Abcam (Cambridge, UK). Antibodies for total ERK1/2, ERK1/2 phosphorylated at Tyr‐204, total p38 MAPK and phosphor (P)‐p38 MAPK were from Cell Signaling Technology (Beverly, MA).

### 2.2 Cells

The mouse retinal microvascular endothelial cells (mRMECs) were cultured in ECM medium supplemented with 10% FBS and 1%EGF and 1%Penicillin/ Streptomycin at 37°C, 5% CO_2_ and 95% air. To study the effect of hyperglycemia, the cells were exposed to normal (physiological) glucose (NG) (5.5 mM), HG (25.0 mM) concentrations, or mannitol (5.5 mM glucose +19.5 mM mannitol) as osmotic pressure control (OC).

### 2.3 Animals

*Fpr2* ^−/−^ mice of C57BL/6 background were generated as described previously.^23^ Littermates derived from Fpr2^+/−^ heterozygotes were used in this study. Care and treatment of animals were in agreement with the guidelines of the Association for Research in Vision and Ophthalmology Statement for the Use of Animals in Ophthalmic and Visual Research and approved by the institutional Animal Care and Use Committees in Affiliated Hospital of Nantong University.

### 2.4 Diabetic mouse model

Eight‐week‐old C57BL/6 J mice (male, n = 15) and *Fpr2* ^−/−^mice (male, n = 15) were given five consecutive intraperitoneal injections of streptozotocin (STZ; 60 mg/kg body wt/day) (Sigma‐Aldrich, St. Louis, MO). Control received citrate buffer alone. At 1 week after STZ injection, hyperglycemia was confirmed in tail prick blood samples using Blood glucose monitoring system (LifeScan, CA, USA). Blood glucose values ≥16.7 mmol/L were considered as diabetic and the mice were enrolled into the study. The blood glucose and weight were monitored before and after STZ injection at 1, 4 and 12 and 24 weeks. After injection for 24 weeks, one mouse failed to reach the diabetic standard in diabetic group, two mice failed in *Fpr2* ^−/−^ diabetic group. Twelve mice were survived in diabetic group and 12 mice *Fpr2* ^−/−^ diabetic group. The mice were euthanized to collect retinas for retinal flat‐mount immunofluorescence staining (six retinas), frozen tissue sections immunofluorescence staining (six eyes), RT‐PCR (six retinas), and Western blot (six retinas) experiments.

### 2.5 Oxygen‐induced retinopathy (OIR)

OIR model has been used as an animal model of ischemia‐induced retinal neovascular disease.^24^ C57BL/6 J and *Fpr2* ^−/−^ mice on postnatal day 7 (P7) with their nursing mothers were used in an airtight incubator ventilated by a mixture of oxygen and air at a final oxygen concentration of 75 ± 5%. Oxygen was returned to normal level at P12 days postpartum. Untreated WT mice were used as normal controls. To study the effect of the Fpr2 agonist CRAMP, mice were intravitreally administered with 1 μl CRAMP dissolved in phosphate‐buffered saline (PBS) at P12. At P17, whole retinal mounts were isolated and stained with isolectin GS‐IB4. Neovascular area (NV) and vaso‐obliteration area (VO) were analyzed using Image J. Mouse retinas were also isolated for H&E staining, Western blot, and RT‐PCR.

### 2.6 Immunofluorescence staining

After euthanizing, the eyes (n = 6 mice per group) were immediately enucleated and were dissected into two group. For one group, the eyes were fixed by immersing in freshly prepared 4% paraformaldehyde for 10 minutes at 4°C then washed in PBS, then retina was removed from the eyecup for flat‐mount staining. The other group of the eyes were fixed in freshly prepared 4% paraformaldehyde for 10 minutes at 4°C, then embedded in optimal cutting temperature (OCT) medium and cryosections were cut at 10 mm for frozen tissue sections.

Frozen tissue sections were reheated and washed with PBS for three times, and then treated for 2 hours at room temperature with 0.05% Triton x‐100 and 3% BSA. The sections were incubated with primary antibodies overnight at 4°C. After washing with PBS for three times, the sections were incubated with corresponding secondary antibodies at room temperature for 2 hours. After staining with DAPI to visualize nuclei, the sections were analyzed under a fluorescence microscope (Zeiss 510; Carl Zeiss, Germany).

Retinal flat‐mount staining and quantification analysis were conducted as previously described.^25^ Endothelial cells proliferation was conducted as previously described.^26^ After fixed in 4% PFA, the retinas were dissected and flattened with four radial incisions. After being rinsed and blocked for non‐specific reactions, the dissected and whole retinas were stained with GS‐IB4 and anti‐Collagen IV/ KI67 overnight at 4°C, then incubated with corresponding secondary antibodies at room temperature for 2 hours.

### 2.7 Real‐time quantitative RT‐PCR

Total RNA from retinal tissues was extracted by using the RNeasy Mini Kit (QIAGEN) and reverse transcribed to cDNA by using cDNA first‐strand synthesis system (Thermo, America). Real‐time PCR was performed on an Applied Biosystems StepOne Real‐Time PCR System. Mouse sequence specific primers for Fpr2, interleukin (IL)1‐β, tumor necrosis factor‐α (TNF‐α), IL‐6, and IL‐10 were synthesized by Invitrogen Biotechnology Co. Ltd, (Shanghai, China). Thermal cycling conditions included an initial denaturation at 50°C for 2 minutes, 95°C for 10 minutes, 40 cycles at 95°C for 15 seconds, 60°C for 30 seconds. The level of mRNA expression in each sample was determined after correction with GAPDH. Changes in relative expression of the genes were calculated by comparing the CT values. Sequence specific primers were as follows: Fpr2 forward: 5′‐CCTGGCCCAAAACATAG‐3′ and reverse: 5′‐ACAGCAGTTGCTTCCTT‐3′. IL1‐β forward: 5′‐GAAGAAGAGCCCATCCTCTGT‐3′ and reverse: 5′‐TGTTCACGGAGCCTGTAG‐3′. TNF‐α forward: 5′‐CTCCACTTGGTGGTTTGCTAC‐3′ and reverse: 5′‐CTTCCCTCTCATCAGTTCTATGG‐3′. IL‐6 forward: 5′‐ACCACTCCCAACAGACCTGTCT‐3′ and reverse: ‐3′ and reverse: 5′‐CAGATTGTTTTCTGCAAGTGCAT. IL‐10 forward: 5′‐ACCTGGTACAAGTGATGCC‐3′ and reverse: 5′‐CAAGGAGTTGTTTCCGTTA‐3′. GAPDH forward 5′‐TGAGCAAGAGAGGCCCTATC‐3′ and reverse 5′‐AGGCCCCTCCTGTTATTATG‐3′.

### 2.8 Western immunoblotting

Retinas were lysed with sample buffer, sonicated and then heated at 100°C for 5 minutes. Total protein was separated by electrophoresis in 10% sodium dodecyl sulfate polyacrylamide gel (SDS‐PAGE) and transferred to PVDF membrane. PVDF membrane was then sealed in 5% skim milk at room temperature for 2 hours and incubated with primary antibodies at 4°C overnight. The membranes were then washed with TBST for three times and incubated with corresponding secondary antibodies at room temperature for 2 hours. Immunoreactive bands were visualized using an enhanced chemiluminescence system and analyzed by Image J.

### 2.9 H&E staining

Eyes were fixed by immersing in freshly prepared 4% paraformaldehyde for 10 minutes at 4°C, then embedded in OCT medium and sagittally sectioned parallel to the optic, then stained with HE. Preretinal neovascular cell nuclei were quantified as previously reported.^27^

### 2.10 Statistical analysis

Results were expressed as the mean ±SE. GraphPad Prism (v6.0, GraphPad Software Inc.) was used for statistical analyses. ANOVA was conducted to compare the differences in results obtained from mouse groups and post hoc multiple pairwise comparisons were performed using Tukey multiple comparison test. A value of *p* < .05 was considered as statistically significant.

## 3 RESULTS

### 3.1 Characterization of STZ‐induced diabetes in mice

Diabetic WT mice showed a body weight loss of 25.11% compared with age‐matched control mice after 6 months. The loss of body weight shown by diabetic *Fpr2* ^−/−^ mice was 19.95% compared with non‐diabetic controls. The blood glucose level in diabetic WT mice increased by 4.01‐fold compared with control mice. In diabetic *Fpr2* ^−/−^ mice, blood glucose increased by 3.31‐fold, compared to non‐diabetic control mice (Figure S1). Therefore, *Fpr2* ^−/−^ mice showed milder diabetic conditions as compared with WT diabetic counterparts.

### 3.2 Increased Fpr2 expression in the retina of diabetic mice

**Figure 1:**
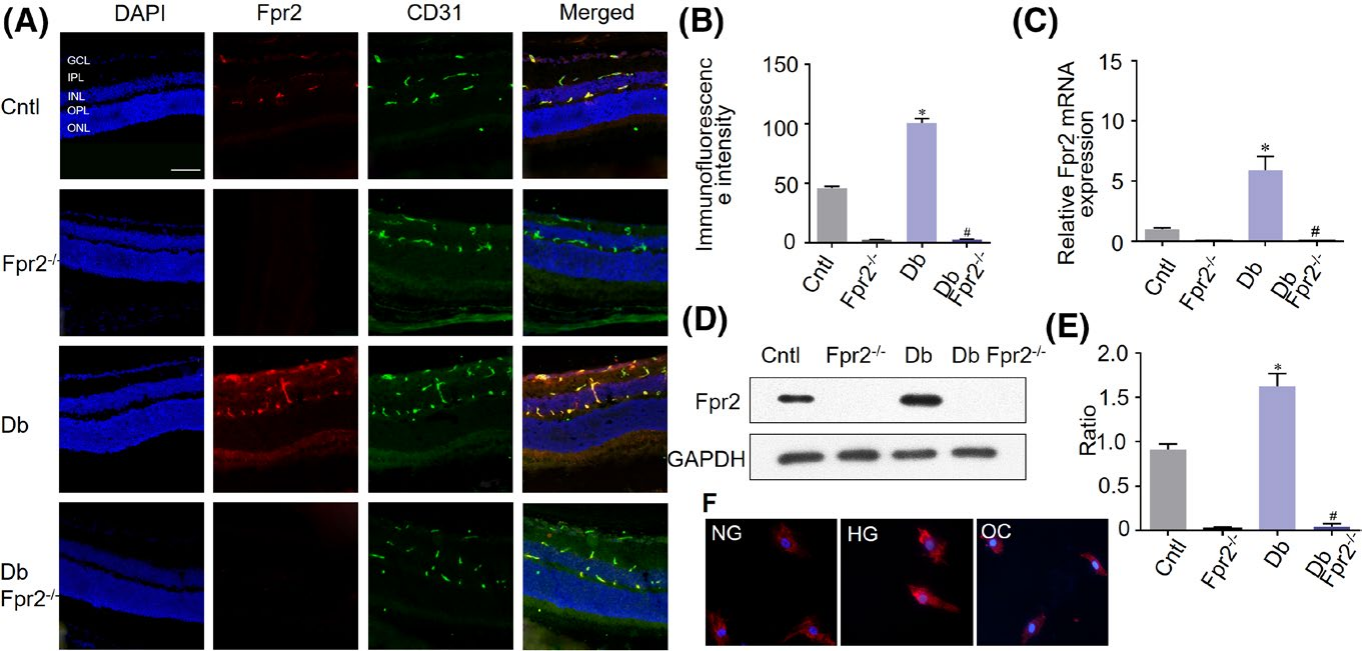
The expression of Fpr2 in mouse retinas with STZ‐induced diabetes. *Fpr2* ^−/−^ mice and WT littermates were treated with STZ to induce diabetes. Control mice were injected with citrate buffer. A, Immunofluorescence staining of Fpr2 (red), CD31 (green) and nuclei (blue) in the retinas of C57BL/6 J (Cntl), *Fpr2* ^−/−^ (*Fpr2* ^−/−^), diabetic WT (Db), and diabetic *Fpr2* ^−/−^ (Db *Fpr2* ^−/−^) mice. Scale bar: 75 μm. B, Fpr2 immunofluorescence intensity quantified based on images in (A). * indicates significantly (*p* < .05) increased Fpr2 intensity in Db WT mouse retinas compared with normal mouse retinas. # indicates the absence of Fpr2 fluorescence in Db *Fpr2* ^−/−^ mouse retinas compared with Db WT retinas. C, Fpr2 mRNA in each mouse group. * indicates significantly increased (*p* < .05) Fpr2 mRNA in Db WT mouse retinas compared with normal mouse retinas. # indicates the absence of Fpr2 mRNA in Db *Fpr2* ^−/−^ mouse retinas compared with Db WT mouse retinas. D, Western blotting showing Fpr2 in protein the retina of mice. E, Densitometry quantification of Fpr2 protein. Results are presented as fold changes. * indicates significantly increased (*p* < .05) Fpr2 protein in Db WT mouse retinas compared with normal mouse retinas. # indicates the absence of Fpr2 protein in Db *Fpr2* ^−/−^ mouse retinas compared with Db WT mouse retinas. F, Immunofluorescence staining of Fpr2 (red) in mRMECs

In normal mouse retina, Fpr2 was expressed in ganglion cell layer (GCL), inner plexiform layer (IPL), and outer plexiform layer (OPL), consistent with vessel locations (Figure 1A). Double immunofluorescence staining with Fpr2 and CD31, a marker for vascular endothelial cells, showed that Fpr2 was mostly expressed by retinal vascular endothelial cells (Figure 1A, B). The expression of Fpr2 mRNA and protein was markedly upregulated in the retina of STZ‐treated diabetic WT mice, with increased proliferation of vascular endothelium, both of which were absent in *Fpr2* ^−/−^ mice (Figure 1C‐E). Figure 1F shows that mRMECs exposed to normal glucose concentration expressed a low level of Fpr2, which was significantly increased under high glucose condition. Additional tests showed that OC has no effect on Fpr2 expression in mRMECs.

### 3.3 Reduced glial cell dysfunction in diabetic *Fpr2* ^−/−^ mice

**Figure 2:**
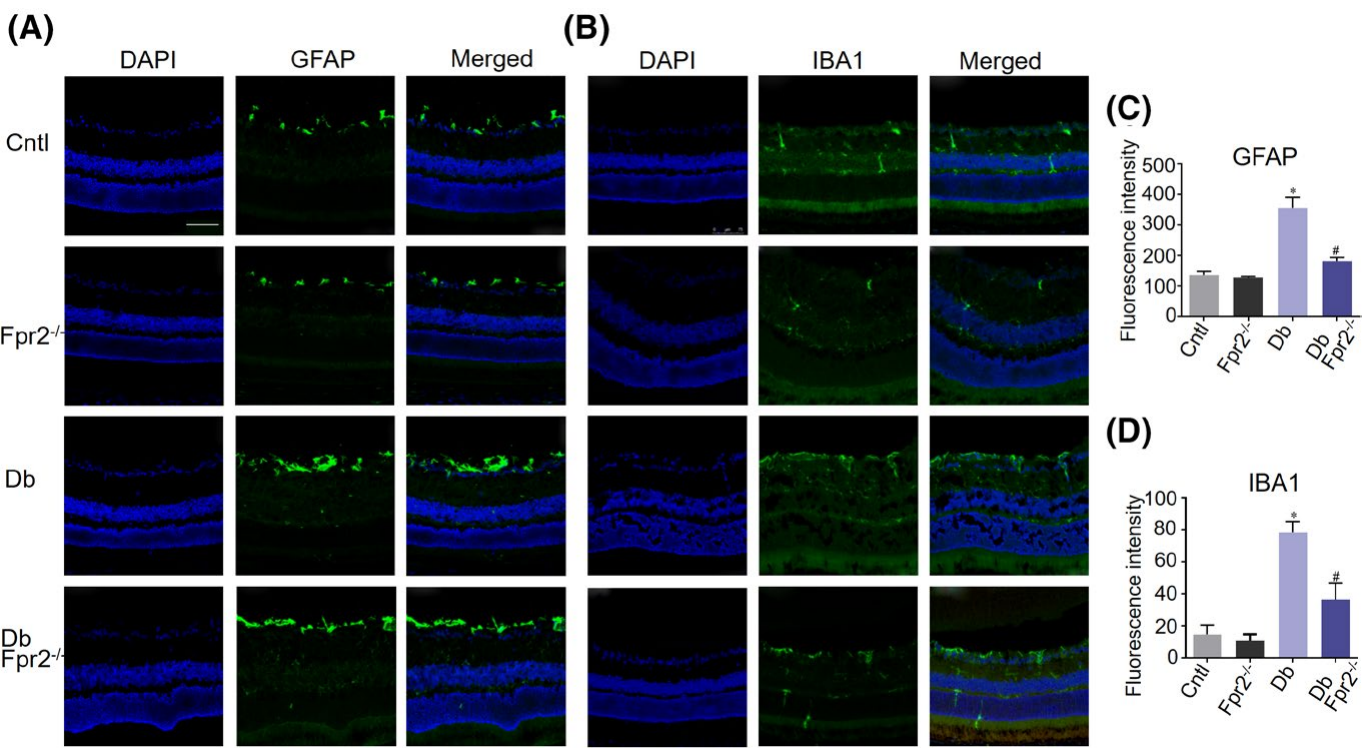
The expression of glial markers in mouse retinas with STZ‐induced diabetes. Immunofluorescence staining of GFAP and IBA1 in retinas of WT C57BL/6 J (Cntl), *Fpr2* ^−/−^ (*Fpr2* ^−/−^), diabetic WT (Db) and diabetic *Fpr2* ^−/−^ (Db *Fpr2* ^−/−^) mice. Scale bar: 75 μm. A, GFAP staining of the retinal Müller glial cells and astrocytes. B, IBA1 staining of the retinal microglial cells. C, GFAP immunofluorescence intensity quantified based on images in (A). * indicates significantly (*p* < .05) increased GFAP intensity in Db WT mouse retinas compared with normal mice retinas. # indicates significantly (*p* < .05) decreased GFAP intensity in Db *Fpr2* ^−/−^ mouse retinas compared with Db WT mouse retinas. D, IBA1 immunofluorescence intensity quantified based on images in (B). * indicates significantly (*p* < .05) increased IBA1 intensity in Db WT mouse retinas compared with WT mouse retinas. # indicates significantly (*p* < .05) decreased IBA1 intensity in Db *Fpr2* ^−/−^ mouse retinas compared with Db WT mouse retinas

GFAP as a marker for glial cells is expressed by retinal astrocytes and Müller cell.^5^ Diabetes significantly upregulated the level of GFAP, concomitant with exacerbated reactive gliosis. In *Fpr2* ^−/−^ diabetic mice, the gliosis was attenuated as indicated by reduction in the intensity of GFAP staining, mostly in the innermost retinal layers (Figure 2A,C). Diabetes also more significantly upregulated the number of Vimentin positive Müller glia cells in WT diabetic mice, as compared to diabetic *Fpr2* ^−/−^ mice (data not shown). Diabetes is also associated with an increase in the number of IBA1 positive retinal microglia in the neuropile, especially within IPL and OPL. IBA1 level was significantly attenuated in *Fpr2* ^−/−^ mice with diabetes indicating reduced microglial accumulation and proliferation in the retina (Figure 2B,D). Thus, STZ‐induced diabetes increased the activation of retinal glia cells including microglia and Müller cells in WT mice. However, Fpr2 deficiency significantly reduced glial cell activation in the retina.

### 3.4 Reduced expression of proinflammatory cytokine mRNA in the retina of diabetic *Fpr2* ^−/−^ mice

**Figure 3:**
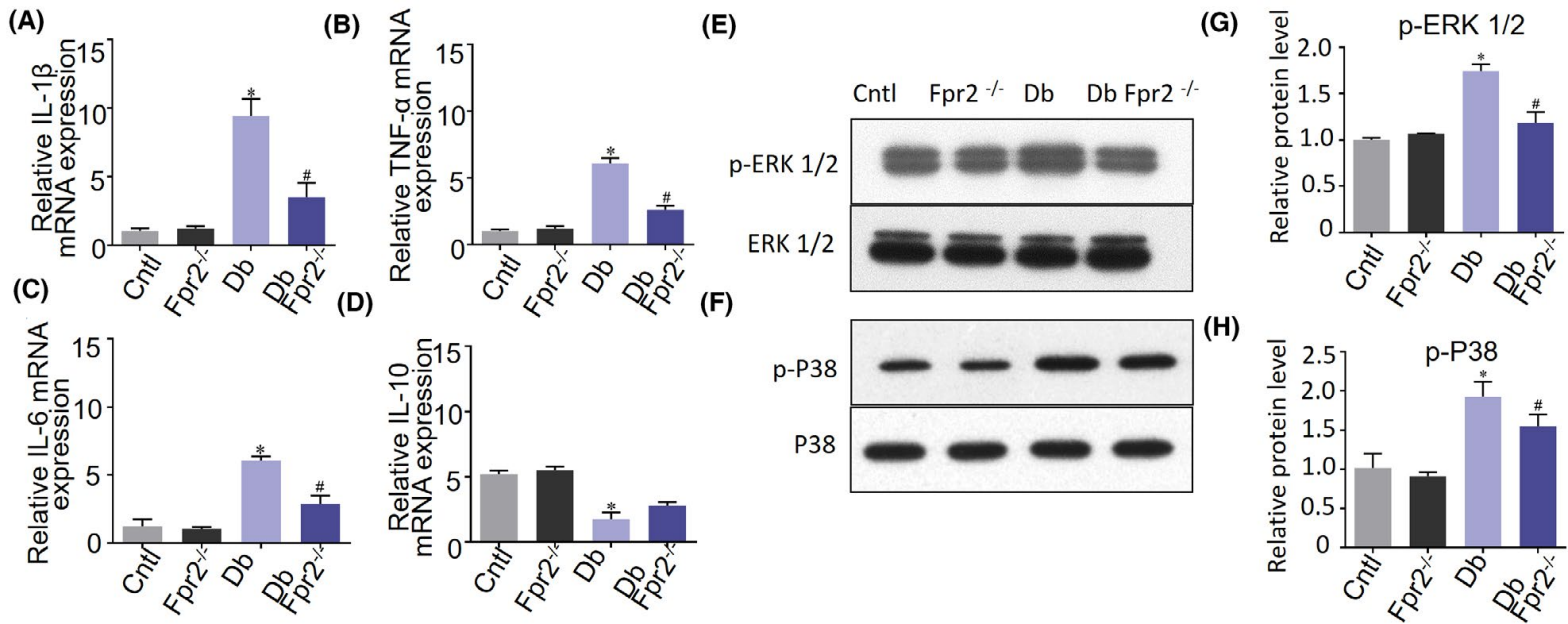
Inflammatory cytokine mRNA and MAPR phosphorylation in the retinas of diabetic mice. A‐D, Cytokine mRNA expression in diabetic mouse retinas measured by RT‐PCR. Histograms represent quantification of mRNA expression. The average value for each sample was normalized against the amount of GAPDH. Cytokine mRNA measured includes IL‐1β, IL‐6, TNF‐α, and IL‐10. * indicates significantly (*p* < .05) increased mRNA levels in diabetic WT mice. # indicates significantly (*p* < .05) decreased mRNA levels in diabetic *Fpr2* ^−/−^ mice. E, F, Western blotting performed to examine the phosphorylation of P38 and ERK1/2 in the retinas of diabetic mice. E, ERK1/2 phosphorylation in diabetic mice. F, P38 phosphorylation in diabetes mice. G, Densitometry quantification of ERK1/2 phosphorylation normalized against total ERK. Results are presented as fold changes. * indicates significantly (*p* < .05) increased ERK phosphorylation in diabetic WT mice. # indicates significantly (*p* < .05) decreased ERK1/2 phosphorylation in diabetic *Fpr2* ^−/−^ mice. H, Densitometry quantification of phosphorylated P38 normalized against total P38. Results are presented as fold changes. * indicates significantly (*p* < .05) increased P38 phosphorylation in diabetic WT mice. # indicates significantly (*p* < .05) decreased P38 phosphorylation in diabetic *Fpr2* ^−/−^ mice.

We then examined the expression of genes coding for proinflammatory cytokines in the retina of mice. The expression of mRNA for TNF‐α, IL‐1β, and IL‐6 was significantly elevated in the retina of WT diabetic mice, with significantly decreased levels of the mRNA for the anti‐inflammatory cytokine IL‐10 (Figure 3A‐D). In contrast, in the retina of *Fpr2* ^−/−^ diabetic mice, the expression of the genes encoding proinflammatory cytokines was significantly reduced (Figure 3A‐D).

The increase in the levels of inflammatory cytokine genes was correlated with the activation status of MAPK‐signaling pathway triggered by many stimulants including high glucose and agonists for the GPCR Fpr2. Examination of the retinas from diabetic WT mice revealed a higher level of phosphorylation of ERK1/2 and P38 MAPKs (Figure 3E‐H). In contrast, in the retina from diabetic *Fpr2* ^−/−^ mice, the phosphorylation of MAPKs was significantly diminished. These results indicate an important role of Fpr2 in amplifying high glucose‐mediated inflammatory responses in diabetic retina.

Reduced proliferation of retinal capillaries in diabetic and OIR *Fpr2* ^−/−^ mice.

**Figure 4:**
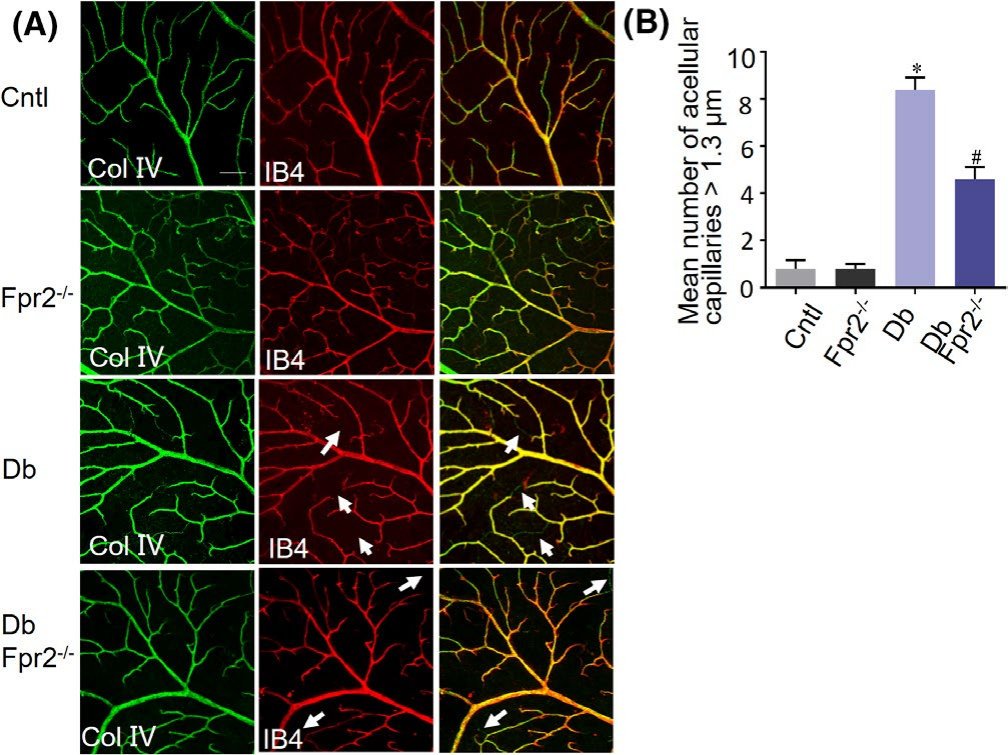
Pathological acellular capillaries in diabetic mouse retinas. Retinal flat mount was analyzed for pathological acellular capillaries. A, The retinal vasculature visualized in flat mounts using concomitant labeling of endothelium (IB4) and basement membrane (collagen IV). Acellular capillaries are visualized by continual collagen IV positivity with loss of endothelium. Images show acellular capillaries (arrows) with collagen IV positive (green) and IB4 negative (red) vessels. B, The number of >1.3 μm acellular capillaries in mouse retinas. * indicates significantly (*p* < .05) increased number in Db WT mouse retinas compared with normal WT mouse retinas. # indicates significantly (*p* < .05) decreased number in Db *Fpr2* ^−/−^ mouse retinas compared with Db WT mouse retinas

**Figure 5:**
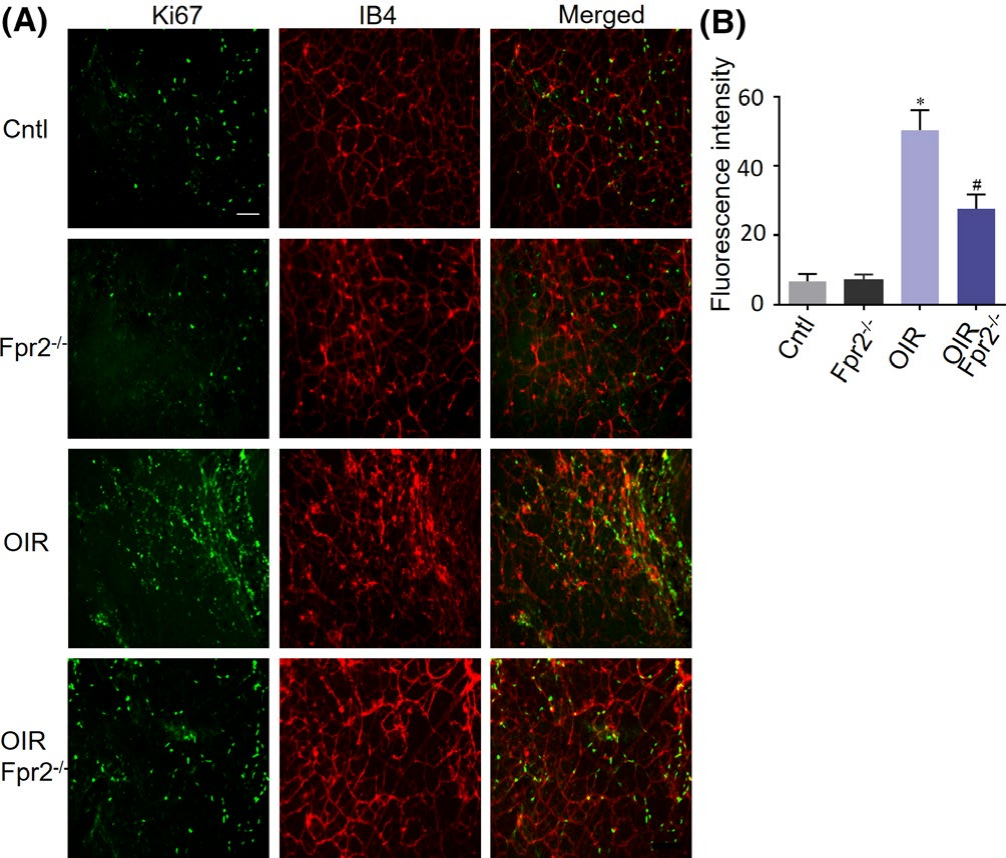
The effect of Fpr2 on cell proliferation in the retinas of OIR mice. Retinal proliferation was visualized in flat mounts by concomitant labeling of endothelium IB4 (red) and Ki67 (green). A, Increased cell proliferation was observed in oxygen‐induced WT mouse retinas than in WT C57BL/6 J (Cntl) mouse retinas. Cell proliferation was significantly reduced in *Fpr2* ^−/−^ OIR mouse retinas. B, Immunofluorescence intensity quantified based on images in (A). * indicates significantly (*p* < .05) increased intensity in Db WT mouse retinas compared with normal mice retinas. # indicates significantly (*p* < .05) decreased intensity in Db *Fpr2* ^−/−^ mouse retinas compared with Db WT mouse retinas

Retinal vasculature is visualized in flat mounts using concomitant labeling of endothelium (IB4) and basement membrane (collagen IV).^28^ Acellular capillaries are visible in the continuance of collagen IV positivity but loss of IB4. Our study shows that the retinas in diabetic WT mice contained approximately sevenfold increase in the numbers of acellular capillaries compared with non‐diabetic retinas (Figure 4A,B). However, there were decreased acellular vessels in the retinas of diabetic *Fpr2* ^−/−^ mice relative to diabetic WT mice (Figure 4A,B). The pathology of OIR bears similarities to DR in mice. In the murine OIR model, Ki67 is used as a marker for neuronal cell proliferation. IB4, as a marker of the vascular network,^29^ was used to label retinal vasculatures. In murine OIR, increased cell proliferation in WT mouse retina was detected by Ki67 and IB4 staining (Figure 5A,B) indicating an increase in endothelium in the ischemic retina of WT mice, with reduction in the ischemic retina of *Fpr2* ^−/−^ mice.

### 3.5 Reduced ischemia‐induced neovascularization in OIR *Fpr2* ^−/−^ mice

**Figure 6:**
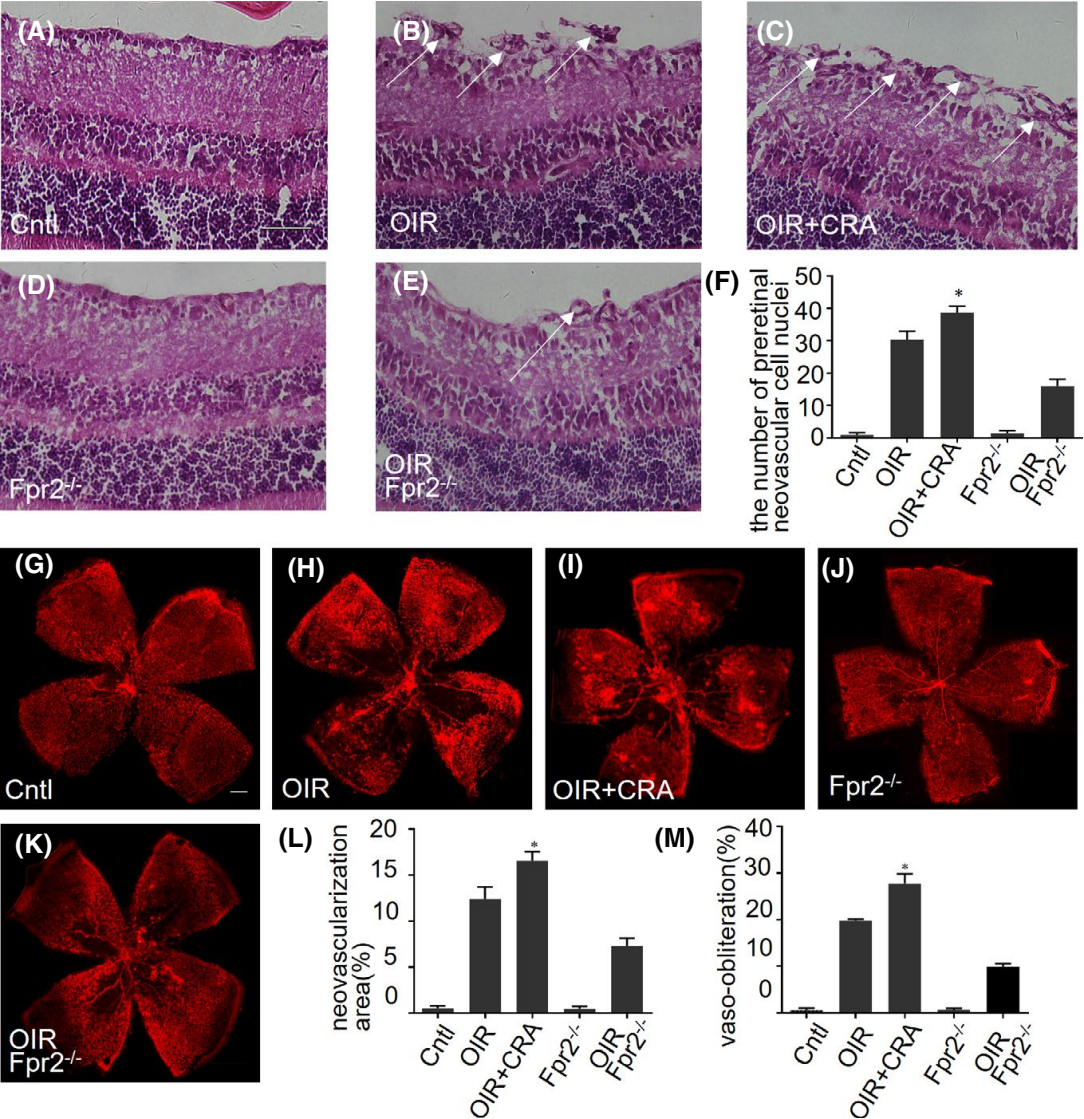
The effect of Fpr2 on pathological angiogenesis in OIR mice. H&E staining was used to detect pathological retinal neovascularization. A‐E, Neovascular cell nuclei anterior to internal limiting membrane (ILM) that represent the extent of retinal neovascularization in the retinas as indicated with arrows. F, The number of preretinal neovascular cell nuclei on the vitreous side of ILM calculated based on (A). *indicates significantly (*p* < .05) increased neovascular cell nuclei in OIR WT mice treated with CRAMP compared with OIR WT mice. The retinal flat‐mount staining with isolectin IB4 was measured to analyze neovascularization (NV) and vaso‐obliteration (VO) areas. G‐K, Images of NV and VO areas in the retinas of each mouse group. L, NV areas quantified based on images in (G‐K). *indicates significantly (*p* < .05) increased NV areas in OIR WT mice treated with CRAMP compared with OIR WT mice. M, VO areas quantified based on images in (G‐K). *indicates significantly (*p* < .05) increased VO areas in WT OIR mice treated with CRAMP compared with WT OIR mice

We further measured retinal neovascularization and ischemic region in the retina of OIR mice by quantification of preretinal neovascular cell nuclei.^27^ H&E staining showed an increased number of preretinal neovascular cells in WT OIR mice (Figure 6A,B), which was significantly reduced in *Fpr2* ^−/−^ OIR mice (Figure 6D,E). After intravitreal injection of the Fpr2 agonist CRAMP (1 μg in 1 μL PBS) in WT OIR mice, the cell number was significantly increased compared to WT OIR mice (Figure 6C), indicating the angiogenic activity of Fpr2 in response to the agonist CRAMP. Retinal flat‐mount labeling showed increased neovascular tufts emerging in WT OIR mice at the boundary between nonperfused and perfused zones at P17 (Figure 6G,H). The neovascular tufts and vaso‐obliteration (VO) areas in the retina of OIR *Fpr2* ^−/−^ mice were markedly reduced compared with OIR WT mice (Figure 6J‐M). After administration of CRAMP (1 μg in 1 μL PBS), the neovascularization and VO areas were significantly increased in OIR WT mice (Figure 6I) indicating the capacity of activated Fpr2 to exacerbate the ischemic damage of the retina.

### 3.6 The expression of Fpr2 agonist CRAMP in OIR mouse retinas

**Figure 7:**
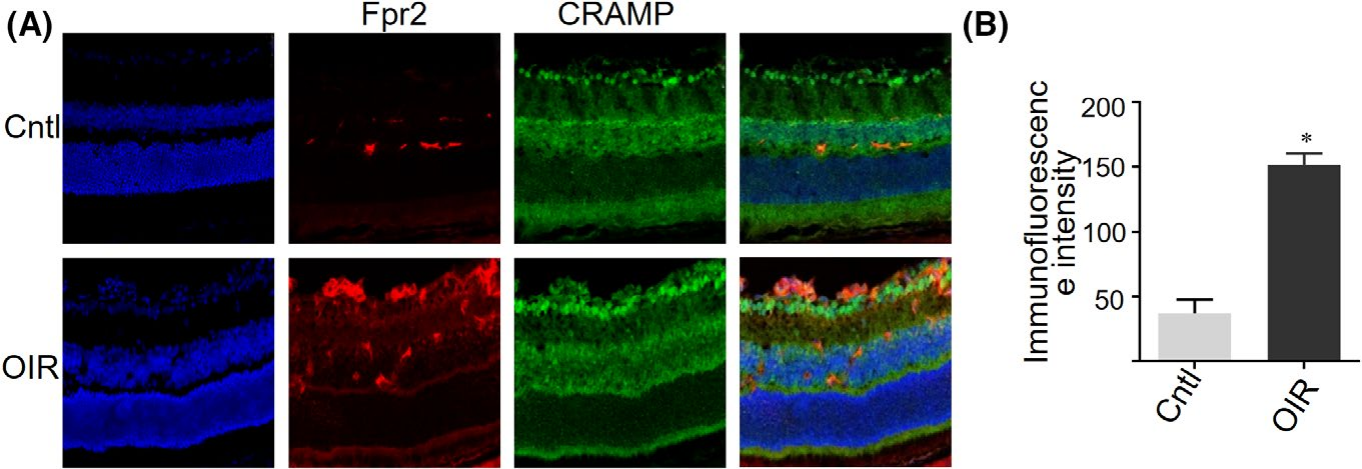
The expression of CRAMP in the retina of OIR mice. CRAMP was detected by immunofluorescence in the retina of OIR mice. A, Increased level of CRAMP in OIR WT mouse retinas compared with normal WT retinas. CRAMP: green, Fpr2: red and nuclei: blue. B, CRAMP immunofluorescence intensity quantified based on images in (A). * indicates significantly (*p* < .05) increased CRAMP intensity in OIR WT mouse retinas compared with normal WT mouse retinas

To investigate the production of potential Fpr2 agonist in the retina, we examined the presence of CRAMP in mouse retinas. Consistent with our previous observations in DR retina, CRAMP level was markedly increased in glial cells from OIR mouse retinas (Figure 7A,B). Interestingly, CRAMP levels were also increased in the *Fpr2* ^−/−^ mouse retinas, supporting the notion for the important role for Fpr2 in mediating the proinflammatory and proangiogenic activity of CRAMP in the eye.

## 4 DISCUSSION

In this study, using two established models for early and late stages of DR, we show that the G‐protein coupled chemoattractant receptor, Fpr2, plays a significant role in exacerbating the pathogenesis of the diseases, which include increased activation of retinal glia cells, retinal inflammatory responses, and angiogenesis.

Our study demonstrated that all three cell types of retinal glia responded rapidly reactivity to hyperglycemia anteceding increased vessel permeability. Because of their close topological association with vessels, retinal glial cells are major targets of vascular alteration and act as communicators between vessels and neurons during disease progression.^30^ Reactive Müller cells are involved in the regulation of vascular permeability and neuronal apoptosis in DR.^31^ Astrocytes appear to be pivotal in ion homeostasis, neuronal signaling, and the integrity of retinal endothelial barrier. In DR, activated astrocytes display increase proliferation, migration, hypertrophy, and secretion of proinflammatory cytokines such as IL‐6 and chemokines.^32^ In diabetic retinas, activated microglia mediate inflammatory responses resulting in further recruitment of leukocytes that cause vascular breakdown, glial dysfunction, and neuronal cell death.^33^, ^34^ Retinal glial cells are also activated under ischemic conditions in OIR.^35^, ^36^ Activated astrocytes produce VEGF and other cytokines, important for revascularization to maintain oxygen supply.^37^, ^38^

Our study discovered a pivotal role of the GPCR Fpr2 in promoting the pathogenesis of DR and OIR, as demonstrated by significantly ameliorated disease processes severity in mice with Fpr2 deficiency, in which the upregulation of GFAP, IBA1, and Vimentin level seen in the diseases was significantly attenuated in *Fpr2* ^−/−^ mice with diabetes, in association with reduced glial activation and accumulation in the retina. The absence of Fpr2 also attenuated the expression of the proinflammatory cytokines in the diabetic retina. The involvement of Fpr2 in the disease progression was confirmed by FPR inhibitor UPARANT to abolish inflammatory responses seen in human PDR vitreous implicating in the role of human FPRs in the regulation of innate immune responses, inflammation, and angiogenesis in the eye.^21^ Fpr2 was also shown to mediate Aβ1‐42 activation of glial cells in the brain to exacerbate proinflammatory responses in the brain of Alzheimer's disease (AD).^39^ Thus, Fpr2 in glial cells, which is upregulated by a number of proinflammatory stimulants including TLRs and inflammatory cytokines and furthermore, by high glucose, plays an important role in neuroglial disease conditions including AD, DR, and OIR.^40^

The proinflammatory property of Fpr2 was further verified in our current study of retinal neovascularization, a pathologic process basis of diabetic and ischemic retinopathy.^41^ Neovascularization is a complex process involving multiple molecules. Accumulating evidence shows that FPRs play a crucial role in angiogenesis. In rheumatoid arthritis, a human FPR2 ligand serum amyloid A (SAA) promotes vascular cell proliferation.^42^ IL‐1β increases the expression of FPR2 in microvascular endothelial cells to endothelial cell proliferation.^43^ FPR2 agonist WKYMVm peptide promotes the proliferation, migration, and lumen formation of endothelial progenitor cells through FPR2, thereby promoting angiogenesis and neovascularization restores blood circulation to reduce the degree of tissue damage.^44^ In addition, FPR inhibitor UPARANT prevents ocular angiogenesis and reduces the levels of inflammatory mediators in mice with OIR and laser‐induced choroidal neovascularization.^45^, ^46^ Our previous study showed that high glucose stimulates the expression of Fpr2 in mouse Müller cells, and in concert with b‐FGFR mediate cell, proliferation, and release of VEGF.^22^ Thus, Fpr2 actively participates in the angiogenic processes which are not only beneficial for tissue restoration after injury but also may promote detrimental angiogenesis seen in diabetic retinal diseases as shown in our studies.

Among endogenous chemoattractant ligands recognized by FPR2, LL‐37 is a human cationic peptide derived from the cathelicidin hCAP‐18.^47^ The mouse homolog of LL37 is CRAMP, an important effector molecule of the innate immune system that induces leukocyte chemotaxis and activation.^48^, ^49^ CRAMP controls normal mouse colon epithelial growth, repair and protection against inflammation‐associated tumorigenesis.^50^ CRAMP also directly activates vascular endothelial cells to promote cell proliferation and form vascular‐like structures.^51^ Likewise, human LL‐37 promotes the proliferation and tubule form of umbilical vein endothelial cells through FPR2.^52^ In this study, CD31 and Fpr2 double positive vascular endothelial cells were detected in mouse retinal blood vessels with increased CRAMP production in the retinas of DR and OIR mice. Furthermore, after intravitreal injection of CRAMP in OIR mice, neovascularization and vaso‐obliteration areas were significantly increased indicating the proangiogenic function of CRAMP through Fpr2. These results represent a novel discovery of Fpr2 in RNV as a driving molecule for angiogenesis associated with vascular endothelial cell activation in ischemic retinas. Thus, Fpr2 participates in the pathogenesis of early and late sight‐threatening stages of DR, and harnessing Fpr2 activity may revolutionize the development of therapies.

## AUTHOR CONTRIBUTIONS

H. Chen, J. M. Wang, and H. Guan designed the research. Y. Yu, S. Xue, S. Wang, and S. Liu performed the research. R. Zhu, K. Chen, and X. Cheng analyzed the data. Y. Le contributed new reagents or analytic tools. Y. Yu and S. Xue wrote the paper.

## Supporting information

Supplementary Material
