# Supplementary material for: The G‐protein‐coupled chemoattractant receptor Fpr2 exacerbates neuroglial dysfunction and angiogenesis in diabetic retinopathy
Source: FASEB Bioadv. 2020 Sep 15;2(10):613–23. doi: 10.1096/fba.2020-00034 (PMC7566047; doi:10.1096/fba.2020-00034)
Supplement: Supplementary file 1 — Supplementary Material [file FBA2-2-613-s001.pdf]

Supplementary Data

The G-protein-coupled chemoattractant receptor (Fpr2) exacerbates neuroglial dysfunction and angiogenesis in diabetic retinopathy

Ying Yu, Shengding Xue, Keqiang Chen, Yingying Le, Rongrong Zhu, Shiyi Wang, Shuang Liu, Xinliang Cheng, Huaijin Guan, Ji Ming Wang, Hui Chen

Supplemental Figure 1

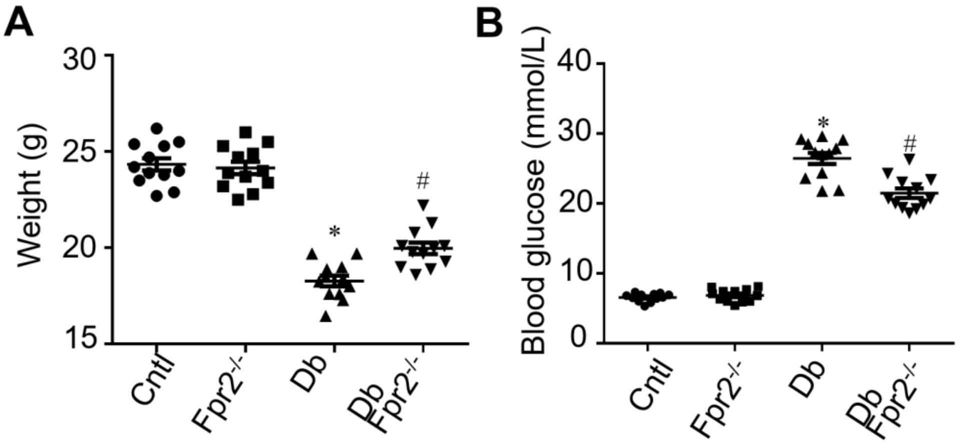

Supplemental Figure 1: Characterization of STZ-induced diabetes in mice

WT and *Fpr2*$^{-/-}$ C57BL/6J mice were injected with STZ to induced diabetes. Mouse body weight and glucose levels in the sera were monitored. (**A**) The body weight of mice treated with STZ or citrate buffer (as control). Results are presented as the mean ± SEM). * indicates significantly decreased ($p < 0.05$) body weight in diabetic (Db) mice compared with mice treated with citrate buffer. # indicates significantly increased ($p < 0.05$) weight in Db *Fpr2*$^{-/-}$ mice compared with WT Db mice. (**B**) Blood glucose levels of mice treated by STZ or citrate buffer. Data are presented as the mean ± SEM). * indicates significantly increased ($p < 0.05$) blood glucose in WT Db mice compared with WT mice treated with citrate acid. # indicates significantly decreased ($p < 0.05$) blood glucose in *Fpr2*$^{-/-}$ Db mice compared with WT Db mice.
